# The FODMAP‐Reducing Potential of Sourdough‐Derived *Lactobacillus* Strains From the Marmara Region of Turkey

**DOI:** 10.1002/fsn3.70241

**Published:** 2025-05-01

**Authors:** Ozen Sokmen, Ayşe Neslihan Dundar, Ufuk Bagci, Sine Özmen Togay, Oya Irmak Sahin, Furkan Turker Saricaoglu

**Affiliations:** ^1^ Department of Food Engineering, Faculty of Agriculture Bursa Uludag University Bursa Turkey; ^2^ Department of Food Engineering, Faculty of Engineering and Natural Sciences Bursa Technical University Bursa Turkey; ^3^ Department of Food Engineering, Engineering Faculty Trakya University Edirne Turkey; ^4^ Department of Chemical Engineering, Faculty of Engineering Yalova University Yalova Turkey

**Keywords:** FODMAPs, irritable bowel syndrome (IBS), lactic acid bacteria (LAB), sourdough, yeast

## Abstract

This study investigates the microbial diversity and FODMAP content of sourdough samples from Turkey's Marmara Region, collected in summer and winter. The primary objective was to identify lactic acid bacteria (LAB) strains capable of reducing FODMAP levels, which are associated with digestive disorders such as irritable bowel syndrome (IBS). The research was conducted in stages; in the first stage, sourdough samples were collected in different seasons and their FODMAP and fructan values were analyzed. In the second stage of the study, LAB strains were isolated from the sourdoughs with the lowest FODMAP and fructan levels, and new sourdough samples were prepared. A comprehensive analytical approach was undertaken, including FODMAP and fructan levels, fermentation characteristics, isolate identification, and chemical analysis of the samples. Among the tested samples, Balıkesir‐1Y‐1 significantly reduced fructan content by 48%, while Tejirdağ‐1 K‐2 and Edirne 1 K‐2 exhibited the highest FODMAP reduction rates of approximately 64%. Additionally, Edirne‐1 K‐2 showed the strongest acidification potential, with 3.76 ± 0.01 pH and 1.20% ± 0.08% total titratable acidity, comparable with the control group. Seasonal variations significantly influenced microbial activity, with summer isolates demonstrating an enhanced enzymatic efficiency in FODMAP metabolism. The results highlight the potential of sourdough fermentation using targeted LAB strains to produce low‐FODMAP foods that maintain high sensory and nutritional quality. This study highlights the significance of optimizing fermentation processes through targeted microbial selection and controlled conditions to achieve desirable nutritional and sensory attributes with dietary sensitivities.

## Graphical abstract

This study investigates the microbial diversity and FODMAP‐reducing potential of sourdough‐derived lactic acid bacteria (LAB) from Turkey's Marmara Region. Key findings reveal that specific LAB strains (e.g., Balıkesir‐1Y‐1, Tekirdağ‐1 K‐2, and Edirne‐1 K‐2) can reduce fructan and FODMAP levels by up to 64%, with extended fermentation decreasing FODMAPs by up to 90%. Seasonal variations influence microbial activity, with summer isolates demonstrating enhanced enzymatic efficiency, supporting the development of low‐FODMAP sourdough for individuals with IBS.

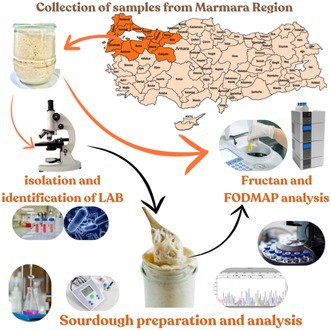

## 1 Introduction

Irritable bowel syndrome (IBS), one of the most common (7%–21%) intestinal disorders worldwide, is characterized by symptoms such as diarrhea, constipation, bloating, and abdominal pain (Giorgio et al. 2015; Marsh et al. 2016). Fructooligosaccharides (fructans) are among the main fermentable oligosaccharides that cause IBS symptoms (Murray et al. 2014). Oligosaccharides, including fructooligosaccharides (FOS), disaccharides, monosaccharides, and polyols, are fermented by colonic bacteria in the intestinal microbiota and are defined as “fermentable carbohydrates” (FODMAPs) (Radosavljević et al. 2024). Given the high prevalence of IBS and the role of FODMAPs in exacerbating its symptoms, dietary choices play a critical role in managing this condition. Whole grain products, despite their significant contribution to dietary fiber intake, pose challenges for IBS patients. Whole grain products included in daily nutrition contribute significantly to dietary fiber intake, but wheat and rye, which are the main components of these products, have high FODMAP content, especially fructans (0.7–2.9 km) (Biesiekierski et al. 2011; Shewry and Hey 2015; Ziegler et al. 2016). This situation limits the usability of whole grain products by IBS patients. In order to address these limitations, alternative fermentation methods, such as sourdough fermentation, have gained attention for their potential to reduce FODMAP levels in food products. Sourdough, a leavening agent, has existed for millennia. It is produced through the fermentation of wheat flour with water, which gives rise to a medium that is enriched with numerous *Lactobacilli* and yeast species (Vogel et al. 2011; Papadimitriou et al. 2019). The increased utilization of sourdough in food production has been attributed to its health‐bringing properties and improvement of taste (Ripari et al. 2016; Atzler et al. 2024). Despite its historical significance and potential health benefits, the impact of sourdough fermentation on FODMAP levels has only been partially explored. Despite the scarcity of research on changes in FODMAP levels in sourdough‐fermented products, several studies have explored this topic (Li et al. 2020; Siyi et al. 2021; Radoš et al. 2022; Kulathunga et al. 2023; Borowska et al. 2023). Most studies on reducing FODMAP content in foods have focused on cereal‐based products. Torbica et al. (2025) analyzed the FODMAP profile of wholegrain pasta and identified fructooligosaccharides (FOS) as the dominant component. Their findings showed that cooking time and water amount significantly influenced the reduction of FODMAPs. Further research on bread production demonstrated that using a combination of yeast and baking powder reduced FODMAP content in wholegrain bread by 78%–81%, while the addition of chia seed gel nearly eliminated it. In a related study, Borowska et al. (2023) investigated the effects of homofermentative lactic acid bacteria (*Lactiplantibacillus plantarum*, *Lacticaseibacillus paracasei*, and *Pediococcus pentosaceus* ) in whole‐wheat sourdough bread, achieving significant FODMAP reductions. These findings highlight the potential of optimizing food processing techniques and incorporating specific bacterial strains to produce low‐FODMAP cereal products.

Moreover, a limited number of studies focused on determining LAB species within starter cultures that effectively reduce FODMAPs (Li et al. 2020; Fang et al. 2021). Most of these studies have been conducted under controlled laboratory conditions using starter cultures, which may not fully reflect the microbial diversity of traditional sourdoughs. Former studies on the FODMAP‐reducing potential of sourdough typically focus on cereal products produced under controlled laboratory conditions, often with the addition of starter cultures. However, this approach may not fully represent the FODMAP‐reducing potential of traditional sourdoughs with diverse microbiotas. Additionally, there is a limited number of studies examining the use of FODMAP‐reducing *Lactobacillus* isolates from sourdough in functional food production.

In this context, to our knowledge, this study represents the first investigation evaluating the FODMAP‐reducing potential of traditional sourdough samples from Turkey's Marmara region and the *Lactobacillus* strains isolated from these samples. The presented study offers an innovative approach to producing low‐FODMAP functional products that may promote gut health.

## 2 Materials and Methods

### 2.1 Collection of Sourdough From Bakeries and Analysis

**FIGURE 1:**
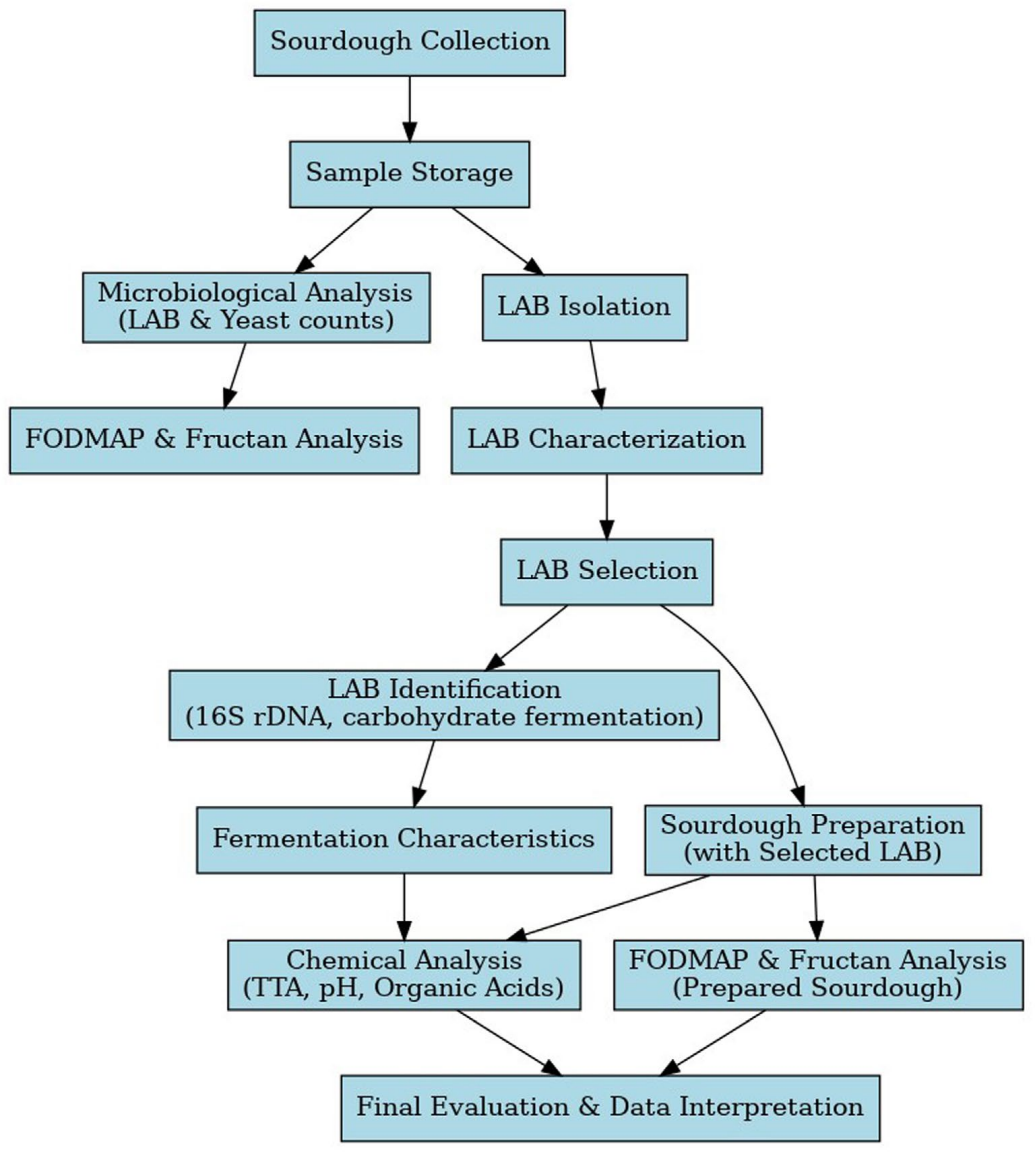
Flow chart.

In the present study, different sourdough samples were obtained from the producers in the Marmara region of Turkey, including Istanbul, Tekirdag, Canakkale, Balikesir, Bursa, Yalova, Kocaeli, Sakarya, Bilecik, and Edirneprovinces. A total of 66 samples were collected during the summer and winter seasons under aseptic conditions, carried in sterile containers, brought to the laboratory and stored at −18°C. The whole wheat flour (Toru Un; Bursa, Turkey) used in the study had 13.10% moisture, 1.38% ash, 13.80% protein, and 32.5% wet gluten. As illustrated in Figure 1, the study was evaluated in two stages. In the first stage, LAB and yeast counts were made in the collected samples, and fructan and FODMAP values of the collected samples were determined. In the 2nd stage, *Lactobacillus* strains were isolated from doughs with low FODMAP values, and the fructose and mannitol usage of these LABs were examined. The most suitable LAB strains were selected from these isolates, and new sourdough samples were prepared and analyzed.

#### 2.1.1 Microbiological Analyses of Collected Sourdoughs

Sourdough samples (25 g) were transferred under aseptic conditions into a stomacher bag containing 225 mL of sterile physiological saline and homogenized. Subsequently, 1 mL of this mixture was taken, and decimal dilutions were made with 9 mL of 0.85% saline solution. For LAB counting and *Lactobacillus* isolation, each sourdough sample was inoculated by surface spreading onto MRS agar (Man, Rogosa and Sharpe, Merck, Germany) containing cycloheximide (0.1% sterile solution, Merck) and incubated anaerobically (Anaerocult A, Merck) at 37°C for 48 h. For yeast counting, inoculations were performed on YGC Agar (Yeast Extract Glucose Chloramphenicol Agar FIL‐IDF, Merck, Germany) using the surface spreading method, and plates were incubated at 25°C for 3–5 days.

#### 2.1.2 Determination of the Amounts of FODMAPs and Fructan in Sourdoughs

Total fructan content was determined spectrophotometrically according to the AACC 32.32.01 method using the Megazyme Fructan commercial enzyme kit (Anonymous. 1999). FODMAPs are a heterogeneous group that includes lactose, glucose, fructose, fructans, and fructooligosaccharides (in trace amounts), galactooligosaccharides (raffinose), and sugar polyols (sorbitol, mannitol). Since high amounts of fructose, glucose, fructans, and mannitol are found in foods, the quantities of these products (fructose, glucose, fructans, mannitol) were determined while measuring the amount of FODMAPs in sourdough collected from different regions and prepared sourdough. Additionally, sourdoughs prepared using the isolated LAB strains were also analyzed for their FODMAP and sugar content. The method provided by Buksa (2020) was applied to determine sugars and sugar alcohols. All FODMAP components, except fructans, were determined using the Agilent‐Infinity 1260 HPLC system. In sugars, an Agilent Hi‐Plex H, 7.7 × 300 mm, 8 μm column and RI detector were used in the HPLC system, and for sugar alcohols, a C18 column and DAD detector were used.

### 2.2 Isolation of LAB Strains, Sourdough Production and Analysis

For *Lactobacillus* isolation, five colonies with different morphologies were selected from the cultures on each plate, transferred onto MRS agar by single‐colony inoculation, and incubated to obtain pure cultures. Gram staining and catalase tests were performed on the pure cultures, and bacteria identified as Gram‐positive, catalase‐negative, and rod‐shaped under microscopic morphology were preserved at −18°C in tubes containing glycerol (30%) and MRS broth.

#### 2.2.1 Sourdough Production With Selected LAB

Sourdough preparations were conducted using FODMAP‐reducing LAB strains with targeted metabolic activity. A 1:1 ratio of wheat flour and water (by weight) was mixed to create a consistency suitable for microbial activity. LAB strains were inoculated at approximately 10^6^ CFU/g, and the mixture was fermented at 25°C for 24 h (Göçmen 2001).

#### 2.2.2 Determination of the Amounts of FODMAPs and Fructan

Total fructan and FODMAP content were determined as described in Section 2.1.

#### 2.2.3 Identification of LAB in Prepared Sourdoughs

The isolates with the potential of FODMAP reduction were identified at the species level by means of 16S rDNA sequence analysis with LacbF/LacbR and LpCoF/LpCoR primer pairs (Minervini et al. 2012; Bartkiene et al. 2020). The PCR products were then sequenced using the Sanger sequencing method, and the obtained sequences were processed for quality control using Chromas v2.6.6. Poor‐quality bases were trimmed, and sequences were manually checked for errors. The edited sequences were then aligned using the ClustalW algorithm in BioEdit v7.2.5.

Selected isolates were also tested for their carbohydrate fermentation. A colony of each culture is spread on MRS medium containing Phenol red (0.05 g/L) as a pH indicator. After inoculation and incubation at 30°C for 24 h, the acid production was indicated by a change of color from red to yellow. The utilized carbohydrates are fructose and mannitol (Rhaiem et al. 2016). Additionally, the growth performance at different temperatures (10°C, 30°C, 37°C, and 45°C) and resistance to acidic conditions (pH 2, 3, 4) were also evaluated (Bell et al. 2005).

#### 2.2.4 Chemical Analysis of Prepared Sourdoughs

*Total titratable acidity* (*TTA*) *and pH*: The pH value was measured using a pH meter, and the acidity was titrated against 0.1 N NaOH. The results were expressed in terms of lactic acid (Aplevicz et al. 2013).

*Determination of organic acids*: For the determination of organic acids, 10 g of sourdough was homogenized in 20 mL of distilled water for 15 min. The mixture was then stirred for 45 min at room temperature in closed tubes. The supernatant was collected after centrifugation at 12000×*g* for 10 min at 4°C, mixed with 0.3 mL of Carrez I (Potassium II hexacyanoferrate, 0.085 mol/L) and 0.3 mL of Carrez II (zinc sulfate, 0.25 mol/L), and vortexed. After centrifugation again, the sample was filtered through a 0.22 μm syringe filter and injected (20 μL) into an HPLC system (Agilent 1200 Infinity, CA, USA). A C18 column (Agilent, Poroshell 120 EC‐C18, 3 mm × 150 mm × 2.7 μm, CA, USA) and a DAD detector (205 nm wavelength) were used. Calibration curves were prepared using organic acid standards in mobile phase at concentrations of 0–100–200–300 ppm, which were used to calculate organic acid concentrations in the samples (Buksa 2020).

### 2.3 Statistical Analysis

Statistical analyses were performed using SPSS (27.0). LSD (Least Significant Difference) test and *t*‐test were used to determine statistically different groups (*p* < 0.05) (Demirkesen‐Bicak et al. 2021).

## 3 Results and Discussion

### 3.1 Microbiological Analyses of Collected Sourdoughs

Yeasts and LAB are microorganisms with significant technological and functional properties, playing a critical role in sourdough fermentation (Chavan and Chavan 2011; Curiel et al. 2015). In this study, total LAB and yeast counts were performed to determine the fermentation activity and microbial load in sourdough samples collected during summer and winter from various provinces of the Marmara Region in Turkey (Table 1). The average LAB count was determined as 6.95 Log CFU/g in summer and 7.87 Log CFU/g in winter. It was observed that the LAB count was generally higher in winter. This increase may indicate that cold weather conditions may be more suitable for some LAB species or that there may be changes in the storage conditions of fermented products (Kou et al. 2022).

**TABLE 1:** Total LAB and yeast count results of sourdoughs collected in summer and winter.

Sample name	Total LAB count (Log CFU/g) summer	Total LAB count (Log CFU/g) winter	Total yeast count (Log CFU/g) summer	Total yeast count (Log CFU/g) winter	pH‐summer	pH‐winter
Balikesir‐1	7.61 ± 0.01	7.20 ± 0.07	6.75 ± 0.05	6.60 ± 0.02	3.82 ± 0.05	4.01 ± 0.05
Balikesir‐2	7.31 ± 0.02	7.35 ± 0.08	7.02 ± 0.02	6.54 ± 0.09	4.03 ± 0.06	3.92 ± 0.05
Balikesir‐3	7.16 ± 0.11	8.15 ± 0.07	6.48 ± 0.02	6.70 ± 0.07	4.19 ± 0.05	3.80 ± 0.05
Bilecik‐1	7.92 ± 0.11	7.20 ± 0.03	< 2.00 ± 0.00	7.41 ± 0.11	3.94 ± 0.05	3.96 ± 0.07
Bilecik‐2	7.90 ± 0.12	7.19 ± 0.05	6.18 ± 0.03	7.19 ± 0.16	3.90 ± 0.08	4.03 ± 0.05
Bilecik‐3	6.19 ± 0.02	< 2.00 ± 0.00	6.15 ± 0.01	6.90 ± 0.15	5.09 ± 0.05	4.37 ± 0.05
Bursa‐1	8.16 ± 0.01	7.15 ± 0.02	6.18 ± 0.05	6.18 ± 0.04	3.96 ± 0.05	4.18 ± 0.08
Bursa‐2	6.62 ± 0.15	< 2.00 ± 0.00	6.11 ± 0.08	7.65 ± 0.04	4.19 ± 0.07	4.24 ± 0.05
Bursa‐3	7.00 ± 0.17	8.70 ± 0.01	6.65 ± 0.07	< 2.00 ± 0.00	3.92 ± 0.05	3.86 ± 0.05
Canakkale‐1	6.18 ± 0.07	8.43 ± 0.11	< 2.00 ± 0.00	< 2.00 ± 0.00	4.66 ± 0.05	3.95 ± 0.06
Canakkale‐2	5.20 ± 0.05	7.31 ± 0.17	4.40 ± 0.07	6.60 ± 0.01	4.60 ± 0.04	4.05 ± 0.05
Canakkale‐3	6.19 ± 0.11	8.32 ± 0.18	5.65 ± 0.09	< 2.00 ± 0.00	5.01 ± 0.05	4.00 ± 0.05
Edirne‐1	7.49 ± 0.19	7.48 ± 0.15	7.02 ± 0.15	5.48 ± 0.16	4.01 ± 0.05	4.14 ± 0.06
Edirne‐2	6.98 ± 0.11	7.61 ± 0.11	5.30 ± 0.19	6.60 ± 0.17	4.01 ± 0.07	3.86 ± 0.05
Edirne‐3	7.27 ± 0.01	7.18 ± 0.07	5.54 ± 0.11	5.70 ± 0.18	4.20 ± 0.05	4.06 ± 0.05
Istanbul‐1	4.00 ± 0.10	< 2.00 ± 0.00	6.36 ± 0.14	7.26 ± 0.02	4.90 ± 0.05	4.22 ± 0.03
Istanbul‐2	6.98 ± 0.12	< 2.00 ± 0.00	7.41 ± 0.02	8.10 ± 0.17	3.90 ± 0.02	4.30 ± 0.05
Istanbul‐3	6.11 ± 0.09	7.74 ± 0.007	< 2.00 ± 0.00	6.30 ± 0.01	4.50 ± 0.05	3.91 ± 0.05
Kirklareli‐1	7.54 ± 0.13	5.98 ± 0.05	4.95 ± 0.01	5.65 ± 0.09	3.98 ± 0.05	4.85 ± 0.09
Kirklareli‐2	7.80 ± 0.19	8.06 ± 0.10	5.62 ± 0.18	7.80 ± 0.10	3.90 ± 0.08	3.78 ± 0.05
Kirklareli‐3	7.36 ± 0.17	6.02 ± 0.02	5.00 ± 0.02	6.36 ± 0.17	4.20 ± 0.05	4.81 ± 0.05
Kocaeli‐1	7.98 ± 0.14	9.58 ± 0.04	5.48 ± 0.05	7.70 ± 0.11	3.92 ± 0.05	3.86 ± 0.07
Kocaeli‐2	6.89 ± 0.07	8.85 ± 0.33	5.48 ± 0.07	7.81 ± 0.15	4.13 ± 0.04	3.85 ± 0.05
Kocaeli‐3	6.80 ± 0.09	10.05 ± 0.20	6.13 ± 0.05	7.30 ± 0.13	4.11 ± 0.05	3.76 ± 0.05
Sakarya‐1	6.44 ± 0.13	9.96 ± 0.10	5.48 ± 0.03	< 2.00 ± 0.00	5.17 ± 0.05	3.94 ± 0.03
Sakarya‐2	6.48 ± 0.17	8.85 ± 0.08	5.61 ± 0.09	6.18 ± 0.02	5.16 ± 0.08	3.93 ± 0.05
Sakarya‐3	6.91 ± 0.19	8.41 ± 0.09	6.83 ± 0.15	< 2.00 ± 0.00	3.91 ± 0.05	3.91 ± 0.04
Tekirdag‐1	6.78 ± 0.02	7.61 ± 0.01	5.98 ± 0.17	< 2.00 ± 0.00	4.01 ± 0.03	3.89 ± 0.05
Tekirdag‐2	6.70 ± 0.01	7.74 ± 0.02	6.11 ± 0.20	< 2.00 ± 0.00	4.03 ± 0.05	3.89 ± 0.08
Tekirdag‐3	6.98 ± 0.07	< 2.00 ± 0.00	6.16 ± 0.04	7.18 ± 0.01	4.08 ± 0.09	4.33 ± 0.05
Yalova‐1	7.63 ± 0.02	7.48 ± 0.06	5.40 ± 0.04	< 2.00 ± 0.00	3.89 ± 0.05	4.19 ± 0.05
Yalova‐2	7.50 ± 0.01	7.32 ± 0.05	5.35 ± 0.01	7.22 ± 0.12	3.97 ± 0.05	4.02 ± 0.07
Yalova‐3	7.21 ± 0.08	7.45 ± 0.09	5.29 ± 0.01	7.04 ± 0.13	4.01 ± 0.06	4.03 ± 0.05

The increase in LAB counts in winter was observed clearly, especially in Kocaeli and Sakarya samples (e.g., an increase from 6.80 to 10.05 Log CFU/g in Kocaeli‐3). The average yeast count was determined as 5.94 Log CFU/g in summer and 6.86 Log CFU/g in winter. A significant increase in the number of yeasts was observed in winter. This increase was especially evident in Bilecik, Kocaeli, and Edirne samples. In some summer samples, yeast counts were recorded as too low to be determined (< 2.00 ± 0.00 Log CFU/g), but a significant increase was observed in winter. For example, while yeast was not detected in Bilecik‐1 in summer, it increased to 7.41 Log CFU/g in winter.

The greatest increase in LAB counts was observed in Kocaeli and Sakarya samples. While this may be partly attributed to lower ambient temperatures and the presence of fermented products during the winter months, it is also important to consider the potential influence of the intrinsic microbiota of the raw materials and variations in storage or handling practices. In samples from Tekirdağ and Yalova, notable differences in yeast counts were observed between summer and winter, which may be related not only to environmental factors such as humidity and temperature but also to the microbial composition inherent to the substrates used. Overall, an increase in both LAB and yeast counts was recorded during the winter season. This trend may result from longer fermentation durations and cooler temperatures that favor the proliferation of these microorganisms. Similar seasonal variations in microbial dynamics have been reported in previous studies (Kou et al. 2022; Liang et al. 2022), highlighting the multifactorial nature of microbial development in fermented foods.

Several studies have investigated LAB and yeast populations in sourdough samples. In these studies, LAB counts were reported to range between 3.0 and 9.59 log CFU/g, while yeast counts varied between 2.0 and 7.92 Log CFU/g (Bakırcı and Köse 2017; Menezes et al. 2019; Tomić et al. 2023; Lai et al. 2023). Microbiologically, LAB are dominant in sourdough, while the yeast content is generally one or two logarithmic cycles lower (De Vuyst et al. 2014; Rizzello et al. 2019). The microbial load of the sourdough varies throughout the sourdough fermentation process. Initially, the microbial load of the sourdough may be comparable to that of the flour. LAB are classified as Gram‐positive or Gram‐negative, and also as aerobic or anaerobic, with the predominant microbial group typically present at cell counts not exceeding 5 Log CFU/g (Onno and Roussel 1994; Stolz 1999; Rocha and Malcata 2012).

LAB are highly characterized by their ability to metabolize mono‐ and disaccharides, resulting in the production of lactic and acetic acids that contribute to the reduction of the pH level in sourdough (Gänzle and Gobbetti 2013). Although the decrease in pH during fermentation can inhibit the growth of some microorganisms, both lactic acid bacteria (LAB) and yeasts exhibit notable acid tolerance. This adaptation enables them to thrive in the sourdough environment despite increasing acidity. Over successive fermentation cycles, these microorganisms progressively adapt and become dominant in mature sourdough ecosystems. However, microbial dominance and metabolic activity can also be influenced by external factors such as temperature and humidity, as well as intrinsic factors including the natural microbiota of the raw materials used. Previous studies have shown that LAB metabolism, particularly the production of organic acids and exopolysaccharides, can vary significantly with temperature (Hammes and Gänzle 1998; Vrancken et al. 2011). For instance, lower temperatures have been associated with slower growth but higher acidification capacity in some LAB strains. In addition, the initial microbial load and species composition in flour and other ingredients contribute to microbial succession during fermentation (De Vuyst and Neysens 2005). Therefore, while seasonal variation may influence microbial counts, it is essential to consider the interplay of these multiple variables to fully understand microbial behavior in sourdough systems. At this point, LAB counts range from 6 to 9 log CFU/g, while yeast counts range from 5 to 8 Log CFU/g (Lattanzi et al. 2013; Minervini et al. 2012).

### 3.2 Determination of the Amounts of FODMAPs and Fructan in Sourdoughs

The analysis of sourdough samples from Turkey's Southern Marmara Region revealed significant seasonal and regional variations in glucose, fructose, mannitol (G + F + M), and fructan levels (Table 2). These variations highlight the influence of microbial activity, fermentation conditions, and environmental factors on sourdough composition.

**TABLE 2:** Total amount of glucose, fructose, mannitol, and fructan (G + F + M) in collected sourdoughs (winter–summer).

Sourdough	G + F + M winter (ppm)	G + F + M summer (ppm)	Fructan winter (ppm)	Fructan summer (ppm)
Control	3502 ± 52^a^	3502 ± 52^a^	1016 ± 112^b^	1016 ± 112^a^
Balikesir‐1	**975 ± 35.28** ^**n**^	**300 ± 30.25** ^**m**^	**216 ± 6.25** ^**no**^	**246 ± 16.24** ^**l**^
Balikesir‐2	1310 ± 67.23^jk^	780 ± 55.78^ijk^	510 ± 4.75^ef^	706 ± 32.70^cd^
Balikesir‐3	1400 ± 77.23^ij^	1444 ± 111.44^ef^	400 ± 10.23^hij^	507 ± 28.46^ghi^
Bilecik‐1	2215 ± 85.32^c^	2579 ± 100.36^b^	506.5 ± 1.75^ef^	467 ± 27.51^hij^
Bilecik‐2	2100 ± 99.86^cd^	1589 ± 89.98^de^	481.5 ± 3.45^f^	624 ± 46.22^defg^
Bilecik‐3	1850 ± 79.81^ef^	1763 ± 79.36^d^	475 ± 5.00^fg^	518 ± 38.32^fgh^
Bursa‐1	610 ± 42.31^o^	683 ± 83.25^jkl^	167.5 ± 1.14^o^	502 ± 22.1^hi^
Bursa‐2	965 ± 63.27^n^	2467 ± 67.39^b^	561.5 ± 8.71^d^	836 ± 36.38^b^
Bursa‐3	1980 ± 88.35^de^	2650 ± 120.35^b^	607.5 ± 5.75^cd^	311 ± 11.31^kl^
Canakkale‐1	1100 ± 84.92^lmn^	883 ± 72.45^ij^	263.5 ± 3.50^mn^	462 ± 22.55^hij^
Canakkale‐2	1201 ± 56.43^klm^	742 ± 42.16^ijk^	300 ± 6.21^lm^	554 ± 24.46^efgh^
Canakkale‐3	1070 ± 57.72^lmn^	1394 ± 75.38^ef^	415 ± 5.41^ghi^	816 ± 36.79^bc^
Edirne‐1	695 ± 36.14^o^	496 ± 46.98^lm^	**161.5 ± 0.71** ^**o**^	**480 ± 25.48** ^**hij**^
Edirne‐2	1500 ± 68.81^hi^	485 ± 65.19^lm^	**155.5 ± 1.41** ^**o**^	**458 ± 38.50** ^**hij**^
Edirne‐3	1730 ± 70.56^fg^	1151 ± 51.28^gh^	321 ± 10.41^lm^	364 ± 28.36^jk^
Istanbul‐1	715 ± 66.78^o^	2544 ± 86.42^b^	342.5 ± 2.50^jkl^	507 ± 27.45^ghi^
Istanbul‐2	**310 ± 48.24** ^**pr**^	**931 ± 91.62** ^**hi**^	447.5 ± 7.54^fgh^	986 ± 36.90^a^
Istanbul‐3	1205 ± 27.21^klm^	1463 ± 66.89^ef^	326.5 ± 6.50^klm^	714 ± 34.36^cd^
Kirklareli‐1	1975 ± 80.85^de^	2238 ± 143.25^c^	1118.5 ± 18.50^a^	726 ± 26.27^bcd^
Kirklareli‐2	1952 ± 79.00^de^	1627 ± 98.46^de^	647.5 ± 24.75^c^	628 ± 68.18^def^
Kirklareli‐3	2135 ± 46.50^cd^	1466 ± 66.36^ef^	500 ± 15.32^ef^	517 ± 17.12^fgh^
Kocaeli‐1	2410 ± 89.91^b^	1478 ± 78.14^ef^	510.5 ± 10.50^ef^	630 ± 20.36^def^
Kocaeli‐2	**990 ± 56.57** ^**n**^	**359 ± 59.35** ^**m**^	207 ± 7.25^no^	459 ± 19.45^hij^
Kocaeli‐3	2440 ± 92.65^b^	942 ± 42.94^hi^	350 ± 15.23^ijkl^	531 ± 21.53^fgh^
Sakarya‐1	735 ± 29.34^o^	945 ± 34.81^hi^	584.5 ± 10.25^cd^	492 ± 12.51^hi^
Sakarya‐2	**185 ± 11.73** ^**r**^	**929 ± 59.27** ^**hi**^	390.5 ± 5.65^hijk^	388 ± 18.76^ijk^
Sakarya‐3	**1855 ± 42.35** ^**ef**^	**453 ± 53.15** ^**lm**^	**498.5 ± 5.80** ^**ef**^	**507 ± 27.38** ^**ghi**^
Tekirdag‐1	407 ± 19.57^p^	1313 ± 86.54^fg^	161.5 ± 2.45^o^	490 ± 40.94^hi^
Tekirdag‐2	1880 ± 68.45^ef^	1596 ± 73.94^de^	297.5 ± 3.41^lm^	510 ± 20.15^gh^
Tekirdag‐3	**260 ± 22.30** ^**pr**^	**1380 ± 80.21** ^**efg**^	**79.5 ± 1.71** ^**p**^	**654 ± 24.43** ^**de**^
Yalova‐1	1645 ± 28.35^gh^	605 ± 35.60^kl^	609.5 ± 15.42^cd^	772 ± 22.55^bc^
Yalova‐2	1015 ± 20.89^mn^	2182 ± 82.14^c^	220.5 ± 3.54^no^	564 ± 34.21^efgh^
Yalova‐3	1230 ± 49.75^jkl^	1821 ± 71.36^d^	297.5 ± 4.50^lm^	658 ± 30.35^de^

*Note:* Data expressed as the mean ± standard deviation. In each line, values followed by different letters are significantly different (*p* < 0.05). The samples highlighted in bold represent those with low fructan and/or FODMAP concentrations, which were selected for further analysis in the subsequent phase of the study.

*Seasonal variations in G + F + M Level*: Among the samples, Balıkesir‐1 exhibited a substantial reduction in G + F + M content, decreasing from 975 ppm in winter to 300 ppm in summer. Conversely, samples like Bursa‐2 and Istanbul‐1 displayed significant increases (*p* < 0.05) during summer, with G + F + M levels rising from 965 ppm to 2467 ppm and from 715 ppm to 2544 ppm, respectively. These contrasting trends suggest that cooler conditions may favor more efficient carbohydrate metabolism in some regions, while others, such as Bursa and Istanbul, may experience increased carbohydrate accumulation due to enhanced microbial activity or substrate availability in warmer months. Regions like Sakarya and Kocaeli, on the other hand, consistently exhibited lower G + F + M levels, indicating a more effective microbial fermentation process regardless of the season.

*Regional differences in fructan levels*: Fructan content demonstrated notable regional and seasonal patterns. For instance, Balıkesir‐2 showed an increase from 510 ppm in winter to 706 ppm in summer, while Tekirdağ‐3 recorded a sharp rise from 79.5 ppm in winter to 654 ppm in summer (*p* < 0.05). In contrast, Bilecik‐1 maintained relatively stable fructan levels across seasons, with 506.5 ppm in winter and 467 ppm in summer, indicating balanced microbial activity. Meanwhile, Bursa‐2 and Canakkale‐3 showed concurrent increases in G + F + M and fructan levels during summer, suggesting an interplay between microbial adaptation and environmental factors favoring carbohydrate retention or slower metabolism.

*High and Low FODMAP Profiles*: Samples such as Kırklareli‐1 consistently exhibited high fructan levels, with 1118.5 ppm in winter and 726 ppm in summer, making it a high‐FODMAP sourdough. In contrast, Balıkesir‐1 and Edirne‐1 maintained relatively low fructan levels, making them potential candidates for low‐FODMAP sourdough production. For instance, Edirne‐1 recorded 161.5 ppm in winter and 480 ppm in summer, reflecting effective fructan reduction during fermentation. Similarly, Sakarya‐3 demonstrated efficient microbial activity, with G + F + M values decreasing from 1855 ppm in winter to 453 ppm in summer, showcasing the potential for optimizing sourdough fermentation for reduced FODMAP content. This study highlights the significant influence of regional and seasonal factors on the fermentation profiles of sourdough samples from Turkey's Marmara Region. Sourdough samples from regions such as Balıkesir‐1 and Edirne‐1 exhibited lower FODMAP content, making them suitable for producing low‐FODMAP sourdoughs. In contrast, regions like Kırklareli and Bursa displayed more variability in their sourdough composition, emphasizing the need for tailored fermentation methods to address diverse microbial and environmental conditions. The results are consistent with previous studies, such as those by Acín Albiac et al. (2020), who demonstrated the effectiveness of fructophilic lactic acid bacteria (FLAB) in breaking down fructans more efficiently than traditional LAB. Similarly, Borowska et al. (2023) found that homofermentative LAB strains like *L. plantarum* FST1.7 and *L. paracasei* R3 are effective in reducing FODMAPs while maintaining product quality. In this study, traditional sourdough samples collected during both winter and summer exhibited notable fluctuations in fructan levels, reflecting the diverse metabolic activities of naturally occurring LAB and yeasts. Notably, samples from Balıkesir‐1 and Edirne‐1 showed the lowest fructan and FODMAP levels, positioning them as potential sources for FODMAP‐reducing LAB isolates. *Lactobacillus* isolation from these samples underscores the value of leveraging local microbial diversity to enhance the nutritional quality of sourdough. These findings reinforce the potential of traditional sourdough fermentation and targeted starter cultures in optimizing sourdough production, particularly for individuals with dietary sensitivities, such as those with irritable bowel syndrome (IBS), while preserving desirable sensory and quality attributes.

The selection of isolates from cities such as Edirne, Tekirdag, İstanbul, Balikesir, Sakarya, and Bursa in the study can be explained by the fact that samples from these regions show a significant reduction potential in both FODMAP and fructan values.

### 3.3 Analysis of Isolated LABs

Table 3 provides information on the characteristics of LAB strains isolated from sourdough samples collected during summer and winter seasons. The isolates exhibiting growth on MRS agar, Gram‐positive staining, and catalase‐negative activity were presumed to be lactic acid bacteria (LAB), as these characteristics are commonly associated with this group. Mannitol zone formation, an indicator of mannitol metabolism, was observed only in two summer isolates, Balikesir‐1Y‐1 and Bursa‐1Y. This suggests that certain LAB strains may have a metabolic adaptation for mannitol production under summer conditions, as most winter isolates did not display this feature.

**TABLE 3:** Characteristics of LAB strains isolated from collected sourdoughs (Winter‐Summer).

Sample name	Mannitol zone formation	Gram staining	Catalase	FODMAPs concentration (glucose, fructose, mannitol ppm)
Balikesir‐1Y‐1	Positive	Gram (+)	Negative	500.6 ± 68^d^
Balikesir‐1Y‐2	Negative	Gram (+)	Negative	226.1 ± 32^d^
Bursa‐1Y	Positive	Gram (+)	Negative	3285 ± 100^c^
Edirne‐1K‐1	Negative	Gram (+)	Negative	9015 ± 215^ab^
Edirne‐1K‐2	Negative	Gram (+)	Negative	8900 ± 160^ab^
Edirne‐1K‐3	Negative	Gram (+)	Negative	8910 ± 230^ab^
Edirne‐1K‐4	Negative	Gram (+)	Negative	8610 ± 100^b^
Istanbul‐2K	Negative	Gram (+)	Negative	8910 ± 150^ab^
Sakarya‐3Y	Negative	Gram (+)	Negative	367 ± 45^d^
Tekirdag‐1K‐1	Negative	Gram (+)	Negative	9230 ± 230^a^
Tekirdag‐1K‐2	Negative	Gram (+)	Negative	8620 ± 100^b^
Tekirdag‐1K‐3	Negative	Gram (+)	Negative	8765 ± 65^ab^
Tekirdag‐1K‐4	Negative	Gram (+)	Negative	8660 ± 100^b^

*Note:* Data expressed as the mean ± standard deviation. In each line, values followed by different letters are significantly different (*p* < 0.05).

### 3.4 Determination of the Amounts of FODMAPs and Fructan of Sourdoughs Prepared With Isolated LAB

In sourdough fermentation, the hydrolysis of fructans is carried out by yeast, while the metabolism of fructans is performed by LAB (Loponen and Gänzle 2018). Therefore, the mannitol consumption abilities of the *Lactobacilli* isolates were evaluated to figure out their fructan‐reducing properties. Fructan and FODMAP concentrations in sourdoughs prepared with different isolates were determined, and the results were compared with the control group (Table 4). The findings revealed that the isolates showed significant differences in terms of their potential to reduce FODMAP content.

**TABLE 4:** Fructan and FODMAP values of sourdough prepared with *Lactobacillus* isolates.

Sample name	Fructan concentration (ppm)	FODMAPs concentration (glucose, fructose mannitol ppm)
Control	612 ± 61^a^	3710 ± 166^a^
Balikesir‐1Y‐1	318 ± 54^c^	2240 ± 156^b^
Balikesir‐1Y‐2	606 ± 45^a^	2320 ± 109^b^
Bursa‐1Y	624 ± 54^a^	980 ± 98^e^
Edirne‐1K‐1	462 ± 16^b^	1490 ± 112^d^
Edirne‐1K‐2	387 ± 33^bc^	1320 ± 176^de^
Edirne‐1K‐3	365 ± 22^bc^	1610 ± 159^d^
Istanbul‐2K	364 ± 36^bc^	2000 ± 122^bc^
Sakarya‐3Y	582 ± 42^a^	2270 ± 20^1b^
Tekirdag‐1K‐2	382 ± 22^bc^	1350 ± 87^de^
Tekirdag‐1K‐3	431 ± 31^bc^	1340 ± 77^de^
Tekirdag‐1K‐4	333 ± 22^c^	1630 ± 96^cd^
Tekirdag‐1K‐1	465 ± 25^b^	1340 ± 159^de^

*Note:* Data expressed as the mean ± standard deviation. In each line, values followed by different letters are significantly different (*p* < 0.05).

In the control group, fructan concentration was measured as 612 ± 61 ppm and FODMAP concentration was measured as 3710 ± 166 ppm. Among the isolates, Balıkesir‐1Y‐1, Tekirdağ‐1 K‐2, and Edirne‐1 K‐2 were determined as the strains that significantly reduced (*p* < 0.05) both fructan and FODMAP levels compared to the control group. Balıkesir‐1Y‐1 isolate reduced fructan concentration to 318 ± 54 ppm (48% decrease) and FODMAP concentration to 2240 ± 156 ppm (40% decrease). Tekirdağ‐1 K‐2 attracted attention by reducing fructan level to 382 ± 22 ppm (37% decrease) and FODMAP level to 1350 ± 87 ppm (64% decrease). Edirne‐1 K‐2 was among the selected strains by reducing the amount of fructans to 387 ± 33 ppm (37% decrease) and the amount of FODMAPs to 1320 ± 176 ppm (64% decrease). On the other hand, Bursa‐1Y isolate provided the highest decrease (*p* < 0.05) in FODMAP concentration to 980 ± 98 ppm (74% decrease), but kept the fructan level at a level similar to the control group at 624 ± 54 ppm.

Extended fermentation times have been shown to significantly reduce fructan levels, emphasizing fermentation as a crucial factor in managing FODMAP content. Studies by Ziegler et al. (2016) and Pejcz et al. (2019) reported fructan reductions of up to 90% through prolonged fermentation, a trend consistent with the results of this study. The findings highlight the importance of fermentation in FODMAP reduction, particularly in sourdough systems where LAB strains actively metabolize complex sugars. By carefully selecting specific LAB isolates and optimizing fermentation times, it is possible to achieve substantial reductions in FODMAP content while maintaining desirable sensory attributes. This research supports the targeted use of LAB isolates as a promising approach for developing sourdough‐based products with reduced FODMAP content. Such innovations could offer significant dietary benefits for individuals with FODMAP sensitivities, including those with IBS.

Selected Balıkesir‐1Y‐1, Edirne‐1 K‐2, and Tekirdağ‐1 K‐2 isolates were determined to be the strains with the highest FODMAP reducing potential due to the significant decrease in both fructan and FODMAP concentrations and were evaluated for further analysis.

### 3.5 Identification of LABs in Prepared Sourdoughs

FODMAP‐reducing LAB strains were isolated from sourdough samples collected from the Marmara Region and identified at the species level using 16S rDNA sequencing. Species identification was performed by comparing the obtained sequences with reference sequences in the NCBI GenBank database using BLASTn. Sequence identity rates were over 98%, and it was determined that the isolates mostly belonged to *Lactiplantibacillus plantarum* and *Lentilactobacillus parabuchneri* species. The obtained results were given in Table 5.

**TABLE 5:** 16S rDNA sequence identification and GenBank comparison results.

Sample Name	Most closely related type strain sequence (GenBank accession no)	Sequence identity (%)	Accession
Balıkesir‐1Y‐1	*Lactiplantibacillus plantarum* strain YFPB1BMX	98.06	FJ538586.1
Edirne‐1K‐2	*Lentilactobacillus parabuchneri* strain NWAFU1163	98.27	MG462056.1
Tekirdağ‐1K‐2	*Lentilactobacillus parabuchneri* strain 24,200	98.00	OR449235.1

The isolates were identified as *L. parabuchneri* (Tekirdağ‐1 K‐2, Edirne‐1 K‐2) and *L. plantarum* (Balıkesir‐1Y‐1) based on sequence similarity. These species are known for their acid tolerance and are commonly used in food fermentation (Van Kerrebroeck et al. 2016; Hüttner et al. 2010). Previous studies have shown the potential of LAB for reducing FODMAP content. Sekwati‐Monang and Gänzle (2011) identified several *Lactobacillus* species, including *Lactobacillus reuteri* and *L. plantarum* , in a sorghum‐based sourdough, and Pejcz et al. (2019) demonstrated that *L. plantarum* could reduce fructan content in wheat bread. Borowska et al. (2023) isolated 244 LAB strains and identified *L. plantarum* FST1.7 and *L. paracasei* R3 as the most effective strains in reducing FODMAPs in whole wheat bread.

In accordance with the results found, sourdough fermentation is primarily driven by LAB, particularly heterofermentative *Lactobacillus* species, which are crucial for the fermentation process (Van Kerrebroeck et al. 2016; Hüttner et al. 2010). While *Lactobacillus* species dominate, other bacterial genera such as *Pediococcus*, *Enterococcus*, *Lactococcus*, and *Weissella* are also present in sourdough (Fujimoto et al. 2019; Yan et al. 2019). Studies have shown that the bacterial population in sourdough typically outnumbers yeast species by a ratio of approximately 100:1 (Arici et al. 2017; Corsetti et al. 1998), highlighting the essential role of bacteria in the fermentation process.

Table 6 showed the resistance of the isolates to acidic conditions was determined in pH 2, pH 3, and pH 4 environments, with the control group (pH 6.5) being taken as a reference. Live cell counts (Log CFU/g) were analyzed at different pH levels. In the pH 2 environment, all isolates decreased to undetectable levels after 3 h (< 2.00 ± 0.00 Log CFU/g) and could not survive. In the pH 3 environment, Balıkesir‐1Y‐1 and Edirne‐1 K‐2 isolates exhibited resistance during the first hour of incubation (Balıkesir‐1Y‐1: 6.30 ± 0.04 Log CFU/g, Edirne‐1 K‐2: 6.78 ± 0.01 Log CFU/g), but decreased to undetectable levels after 3 h. On the other hand, the Tekirdağ‐1 K‐2 isolate demonstrated undetectable levels within the first hour (< 2.00 ± 0.00 Log CFU/g). In the pH 4 medium, all isolates were able to maintain their viability for 3 h and did not show a significant decrease. Notably, Balıkesir‐1Y‐1, Edirne‐1 K‐2, and Tekirdağ‐1 K‐2 maintained viability levels with values very close to their initial cell densities (7.12–7.66 Log CFU/g). These results reveal that Edirne‐1 K‐2 and Balıkesir‐1Y‐1 isolates exhibited short‐term resistance at the pH 3 level, but all isolates lost their viability in long‐term low pH exposure. In the pH 4 medium, all isolates proved the capability of stable survival by validating their capacity to withstand low pH conditions in fermented products.

**TABLE 6:** The growth performance of selected *Lactobacillus* strains at various temperatures.

Kodu	10^ο^C [log(CFU/mL)]	30^ο^C [log(CFU/mL)]	37 ^ο^C [log(CFU/mL)]	45 ^ο^C [log(CFU/mL)]
Balıkesir‐1Y‐1	< 2.00 ± 0.00	7.51 ± 0.14	7.40 ± 0.09	< 2.00 ± 0.00
Edirne‐1K‐2	< 2.00 ± 0.00	7.30 ± 0.14	7.50 ± 0.02	< 2.00 ± 0.00
Tekirdag‐3K‐2	< 2.00 ± 0.00	6.92 ± 0.21	7.63 ± 0.07	< 2.00 ± 0.00

The growth capacities of the isolates at 10°C, 30°C, 37°C, and 45°C were given in Table 7. In the low temperature (10°C) environment, the viable cell counts for all isolates remained at levels that were too low to be determined (< 2.00 ± 0.00 Log CFU/g), indicating that their growth under cold conditions was limited. Contrastingly, at temperatures of 30°C and 37°C, a notably higher growth performance of the isolates was recorded. The isolates Balıkesir‐1Y‐1, Edirne‐1 K‐2, and Tekirdağ‐3 K‐2 exhibited growth rates ranging from 7.30–7.63 Log CFU/g at these temperatures, suggesting that these levels represented their optimal growth temperature. In a high temperature of 45°C, the viable cell counts of all isolates were recorded as too low to be determined (< 2.00 ± 0.00 Log CFU/g), indicating their limited growth ability above 45°C. These results indicate that the isolates showed optimal growth performance within the range of 30°C–37°C, with no growth observed at low temperatures (< 10°C) or high temperatures (> 45°C). These findings demonstrate that the selected LAB isolates under consideration displayed a clear preference for the temperature range of 30°C–37°C, where optimal growth in fermented products was observed.

**TABLE 7:** Resistance characteristics of isolates to low pH environments [log (CFU/mL)].

Sample Name	Live cell count [log (CFU/mL)]											
	pH 2			pH 3			pH 4			Control (pH 6,5)		
	0 h	1 h	3 h	0 h	1 h	3 h	0 h	1 h	3 h	0 h	1 h	3 h
Balıkesir‐1Y‐1	7.18 ± 0.02	< 2.00 ± 0.00	< 2.00 ± 0.00	7.25 ± 0.03	6.30 ± 0.04	< 2.00 ± 0.00	7.48 ± 0.11	7.40 ± 0.14	7.36 ± 0.03	7.40 ± 0.14	7.50 ± 0.09	7.48 ± 0.11
Edirne‐1K‐2	6.37 ± 0.3	< 2.00 ± 0.00	< 2.00 ± 0.00	7.20 ± 0.02	6.78 ± 0.01	< 2.00 ± 0.00	7.55 ± 0.12	7.50 ± 0.02	7.21 ± 0.01	7.50 ± 0.02	7.69 ± 0.21	7.55 ± 0.12
Tekirdag‐1K‐2	< 2.00 ± 0.00	< 2.00 ± 0.00	< 2.00 ± 0.00	5.53 ± 0.08	< 2.00 ± 0.00	< 2.00 ± 0.00	7.66 ± 0.04	7.63 ± 0.07	7.12 ± 0.03	7.63 ± 0.07	7.55 ± 0.03	7.66 ± 0.04

### 3.6 Chemical Analyses of Prepared Sourdoughs

#### 3.6.1 pH and Titration Acidity Analysis in Sourdough Samples

Table 8 presents the pH and total titratable acidity (TTA) results for sourdough samples prepared with various *Lactobacillus* isolates. The control dough, containing only commercial baker's yeast ( *Saccharomyces cerevisiae* ), served as a baseline for comparison. The pH value of the control group was measured as 3.93 ± 0.01 and the TTA value as 0.73% ± 0.21%. Among the isolates, Edirne‐1 K‐2 showed the strongest acidification effect with the lowest pH value (3.76 ± 0.01) and the highest total acidity value (1.20% ± 0.08%) (*p* < 0.05). This result shows that the Edirne‐1 K‐2 isolate is more active in lactic acid production and strongly directs the fermentation process by decreasing the pH. Balıkesir‐1Y‐1 isolate provided higher acidification (*p* < 0.05) compared to the control group by decreasing the pH to 3.89 ± 0.01 and increased the TTA value to 1.10% ± 0.08%. Tekirdağ‐1 K‐2 isolate had the highest pH value of 3.97 ± 0.01 pH, and the TTA value was determined as 1.03% ± 0.05%. Although this value showed a higher acidic (*p* < 0.05) character compared to the control group, it had a milder effect compared to other LAB isolates. In general, LAB isolates showed lower pH and higher total titratable acidity (TTA) values compared to the control group, showing that they were effective acid producers in sourdough fermentation. Edirne‐1 K‐2, in particular, stands out as the isolate with the highest acidification capacity with both the lowest pH value and the highest TTA value (*p* < 0.05).

**TABLE 8:** pH and TTA Analysis of Sourdough Prepared with *Lactobacillus* Isolates.

Isolate code	pH	TTA (%lactic acid)
Control	3.93 ± 0.01^b^	0.73 ± 0.21^b^
Balikesir‐1Y‐1	3.89 ± 0.01^c^	1.10 ± 0.08^a^
Edirne‐1K‐2	3.76 ± 0.01^e^	1.20 ± 0.08^a^
Tekirdag‐1K‐2	3.97 ± 0.01^a^	1.03 ± 0.05^a^

*Note:* Data expressed as the mean ± standard deviation. In each line, values followed by different letters are significantly different (*p* < 0.05).

These results align with earlier studies which identified pH and TTA as major factors in the proliferation of LAB during fermentation (Alkay et al. 2023). For instance, Oshiro et al. (2020) observed that *Lactobacillus* species gradually prevail in the microbial load as pH declines. The Edirne‐1 K‐2 samples flourished at the lowest pH, suggesting its great acid resistance, thus paralleling the succession pattern observed with *L. sanfranciscensis* in traditional sourdough flora (Byun et al. 2025). Also, it has been emphasized that adaptation of LAB to acidic environments through evolutionary selection can enhance acid tolerance. Furthermore, higher acid production correlates with enhanced biochemical properties in sourdough fermentation. Verni et al. (2024) demonstrate higher acidification of LAB strains that increase proteolysis and the release of functional metabolites. Overall, these findings confirm that LAB isolates significantly contribute to the acidification and biochemical complexity of sourdough fermentation.

#### 3.6.2 Determination of Organic Acid Content

Sourdough fermentation results in the formation of acetic acid, lactic acid, and ethyl alcohol due to microbial activity, contributing to its distinctive aroma. Research highlights that organic acids produced by heterofermentative LAB significantly influence the aroma of sourdough products. Lactic and acetic acids are key precursors, alongside smaller amounts of propionic, isovaleric, n‐butyric, and valeric acids. Acetic acid, in particular, enhances both aroma and the effects of other flavor compounds (Göçmen 2001; Jayaram et al. 2013). Succinic acid, lactic acid, and acetic acid concentrations were determined as organic acid content in sourdoughs fermented with different isolates and compared with the control group (Table 9).

**TABLE 9:** Organic acid contents of sourdough prepared with *Lactobacillus* isolates.

Sourdough			
Isolate code	Succinic acid (ppm)	Lactic acid (ppm)	Acetic acid (ppm)
Control	56.4 ± 1.11^a^	9643.0 ± 12.2^a^	692.8 ± 1.55^a^
Balikesir‐1Y‐1	9.6 ± 0.23^b^	2330.7 ± 20.7^b^	242.7 ± 1.07^d^
Edirne‐1K‐2	4.8 ± 0.46^c^	1826.2 ± 12.4^d^	136.9 ± 0.88^e^
Tekirdag‐1K‐2	9.2 ± 1.00^b^	1513.4 ± 11.3^e^	274.7 ± 2.25^c^

*Note:* Data expressed as the mean ± standard deviation. In each line, values followed by different letters are significantly different (*p* < 0.05).

In the control group, succinic acid concentration was measured as 56.4 ± 1.11 ppm, lactic acid as 9643.0 ± 12.2 ppm, and acetic acid as 692.8 ± 1.55 ppm. The high succinic and acetic acid levels in the control sourdough are attributed to the activity of *S. cerevisiae* (*p* < 0.05), which plays a crucial role in the production of these organic acids, impacting flavor development (Jayaram et al. 2013). Edirne‐1 K‐2 exhibited the lowest succinic acid (4.8 ± 0.46 ppm), lactic acid (1826.2 ± 12.4 ppm) and acetic acid (136.9 ± 0.88 ppm) concentrations and showed the lowest organic acid production in comparison with the control group. In contrast, Tekirdağ‐1 K‐2 showed a significant decrease in succinic acid level with values of 9.2 ± 1.00 ppm, lactic acid concentration with 1513.4 ± 11.3 ppm, and acetic acid level with 274.7 ± 2.25 ppm. Balıkesir‐1Y‐1 exhibited a significant decrease in organic acids with succinic acid levels determined as 9.6 ± 0.23 ppm, lactic acid levels at 2330.7 ± 20.7 ppm, and acetic acid levels at 242.7 ± 1.07 ppm. These results show that all LAB isolates significantly decreased succinic, lactic, and acetic acid levels compared to the control group. In particular, Edirne‐1 K‐2 and Tekirdağ‐1 K‐2 isolates stand out with the lowest lactic acid and acetic acid levels (*p* < 0.05), indicating their potential to reduce FODMAP content and alter the organic acid profile of fermented products.

The elevated lactic acid content in the control sample during the initial fermentation is linked to the breakdown of sucrose, fructose, and glucose by yeast into lactic acid (Menezes et al. 2018). When sourdough isolates were used, the profile of organic acids produced varied significantly. A review of organic acid contents in similar studies underscores the variation due to raw material differences, fermentation times, and enzymatic activity during fermentation. For example, acetic acid concentrations reported by Gidari‐Gounaridou et al. (2023) for gluten‐free sourdoughs ranged from 5 to 35 ppm. Other studies reported succinic acid values from 980.74 to 5243 mg/kg and lactic acid values from 4874.74 to 8722.24 mg/kg (De Luca et al. 2021). The variability in organic acid profiles reflects the influence of fermentation conditions and the specific microbiota in sourdough, further confirming the critical role of controlled fermentation in determining sourdough quality.

## 4 Conclusion

The presented study provides novel insights into the microbial diversity and FODMAP‐reducing potential of sourdough samples from Turkey's Marmara Region, which is characterized by diverse climatic and geographical conditions that significantly influenced fermentation dynamics and microbial activity. Samples were obtained from Balıkesir, Edirne, Tekirdağ, Bursa, Kırklareli, Istanbul, Yalova, Kocaeli, Sakarya, Bilecik, and Çanakkale, covering a range of coastal, inland, and transitional climate zones, emphasizing the pivotal role of lactic acid bacteria (LAB) in fermentation processes. The findings emphasize how regional environmental factors, such as temperature and humidity, influence the metabolic activity of lactic acid bacteria (LAB) and, consequently, their ability to reduce FODMAP content, as the Marmara region exhibits diverse climatic conditions. Balikesir, Bilecik, and Çanakkale are located in southern Marmara and have hotter and drier summer seasons, which increases the enzyme activity of LAB. The dominance of *Lactiplantibacillus plantarum* was established in Balıkesir‐1Y‐1 by DNA analysis, which coincided with a profound decrease in fructan concentrations. Edirne and Tekirdağ are northwestern cities of the Marmara region that have colder and more humid winters, supporting the results of microbial acclimation, where samples exhibited the lowest FODMAP concentrations and identified *Lentilactobacillus parabuchneri* as the dominant strain. With more continental climatic influences, Bursa and Kırklareli had lower FODMAP levels in the case of limited fructan degradations. Additionally, the presence of high humidity gave different responses on microbial growth and mechanisms, where it is thought that different microbial interactions occurred for the samples from the cities of Bursa, Yalova, Kocaeli, and Sakarya.

The results support the use of traditional sourdough fermentation combined with targeted microbial selection as a sustainable strategy for enhancing food digestibility and functionality. Future research should focus on expanding the geographical scope of sourdough sampling, exploring advanced fermentation techniques, and optimizing starter cultures to further reduce FODMAP levels while maintaining sensory and nutritional qualities. Ultimately, these findings reinforce the *transformative potential of sourdough fermentation* in improving dietary options for *sensitive consumers*, paving the way for *next‐generation functional foods* that align with *gut health and digestive well‐being*.

## Author Contributions

**Ozen Sokmen:** conceptualization (equal), data curation (equal), methodology (equal), writing – original draft (equal). **Ufuk Bagci:** formal analysis (equal), validation (equal), visualization (equal). **Sine Özmen Togay:** conceptualization (equal), data curation (equal), methodology (equal), supervision (equal). **Oya Irmak Sahin:** formal analysis (equal), validation (equal), visualization (equal). **Furkan Turker Saricaoglu:** writing – review and editing (equal).

## Ethics Statement

The authors have nothing to report.

## Conflicts of Interest

The authors declare no conflicts of interest.

## Data Availability

Data will be made available on request.
